# Size-Dependent Effect of Indocyanine Green Nanoimaging Agent for Metastatic Lymph Node Detection

**DOI:** 10.34133/bmr.0022

**Published:** 2024-04-15

**Authors:** Quoc-Viet Le, Sungtaek Kang, Jaeseong Lee, Hyeseon Park, Jeong Gil Sun, Jaiwoo Lee, Gayong Shim

**Affiliations:** ^1^ Faculty of Pharmacy, Ton Duc Thang University, Ho Chi Minh City, Vietnam.; ^2^ School of Systems Biomedical Science and Integrative Institute of Basic Sciences, Soongsil University, Seoul 06978, Republic of Korea.; ^3^ College of Pharmacy and Research Institute of Pharmaceutical Sciences, Seoul National University, Seoul 08826, Republic of Korea.

## Abstract

Identification of metastatic lymph nodes is a crucial step in lymph node dissection to prevent further cancer spread and recurrence. However, the current limitations in metastatic lymph node detection often result in extensive resection of normal lymph nodes, leading to serious complications. The clinical application of indocyanine green (ICG) as a tool for lymph node detection is challenging because of its short plasma half-life and rapid light-induced decomposition and clearance. To overcome this limitation, we used polydopamine nanoparticles (PNs) as carriers for ICG and screened for the optimal particle size for detecting metastatic lymph nodes. ICG/PNs with sizes of 80, 160, 300, and 600 nm were synthesized, and their ICG loading efficiency, physical stability, and lymph node distribution were evaluated. The ICG absorbed on the PNs was found to be protected from light degradation, and its retention at the lymph nodes was improved. Notably, the ICG/PNs favored the fluorescence signal at the metastatic lymph nodes compared to the nonmetastatic lymph nodes. Among the tested particle sizes, the 80-nm ICG/PN showed a higher distribution in the metastatic lymph nodes. This study suggests that the 80-nm ICG/PN is a potentially valuable reagent for the detection and diagnosis of lymph node metastasis.

## Introduction

The occurrence of lymph node metastasis is a challenge for patients and their healthcare providers. Individuals with lymph node metastases stemming from diverse cancer types frequently face an elevated risk of cancer-related mortality compared to those with cancer-free lymph nodes. Therefore, the presence of lymph node metastasis is a pivotal indicator that determines both the prognosis and appropriate treatment approach for individuals with cancer [1]. Currently, the primary treatment approach to preventing further metastasis is lymph node dissection, in which anatomically suspected lymph nodes are removed. Lymph nodes are important organs that connect lymphatic vessels and generate lymphocytes, aiding the immune system in its defense against pathogens [2]. Extensive resectioning of lymph nodes may cause serious complications, such as lymphedema, due to the blockade of lymph fluid drainage [3]. Preoperative lymph node staging is required to limit the damage caused by extensive lymph node resectioning.

Currently, various imaging agents have been developed to detect and map lymph nodes, including small molecules such as colored and fluorescent dyes and nanoparticles such as radioactive colloids [4]. Among these, indocyanine green (ICG) is an approved imaging agent that is widely used for the assessment of sentinel lymph nodes owing to its biocompatibility and safety profile [5]. However, the use of ICG in near-infrared (NIR)-assisted sentinel lymph node mapping still has limitations regarding its poor photostability due to rapid light-induced degradation and fast clearance. Furthermore, ICG also causes lymph node misidentification because of the nonspecific distribution of small molecules compared to their complex forms with carriers [6]. Various strategies have been studied, including exploiting nanoparticles, such as lipid-based nanoparticles [7] or semiconducting nanoparticles [8], for carrying ICG and protecting it from rapid degradation.

Particle size plays a pivotal role in the migration dynamics of nanoparticles within the lymph nodes. In general, nanoparticles smaller than 100 nm exhibit prompt uptake and prolonged residence within the lymphatic system. However, the ideal size for effective lymph node targeting is contingent upon the specific composition of the nanocarrier material [7,8]. Polydopamine (PDA) has emerged as a promising material in the field of biomedicine because of its straightforward synthesis and excellent biocompatibility. The simple, one-step oxidative polymerization of dopamine under mild conditions is an attractive option for various biomedical applications. Notably, PDA exhibits strong adhesiveness, allowing it to adhere to biological tissues and nanoparticles and provide stability at the interfaces. Furthermore, its surface can be easily functionalized, enabling customization for specific applications. With sizes ranging from approximately 50 to over 600 nm, PDA nanoparticles offer versatility for drug delivery and imaging purposes [9].

In this study, we used PDA nanoparticles (PNs) as carriers of ICG and assessed their utility in NIR fluorescence-assisted lymph node imaging. PNs were synthesized in 4 different sizes of 80, 160, 300, and 600 nm, and ICG was incorporated onto their surfaces, resulting in distinct types of fluorescent nanoparticles. We compared their ICG loading efficiency, physical stability, and photostability. To evaluate the metastatic lymph node imaging efficacy of ICG/PNs of different sizes, we used a luciferase-active orthotopic breast tumor model. Among the various sizes tested, 80-nm ICG/PN exhibited the highest signal accumulation in metastatic lymph nodes. Moreover, we observed selective drainage to metastatic lymph nodes, as opposed to nonmetastatic lymph nodes, in mice treated with 80-nm ICG/PN. This study indicates the potential applications of ICG/PNs for identifying metastatic lymph nodes. ICG/PNs are a valuable tool for preventing the unnecessary removal of healthy lymph nodes during lymph node dissection.

## Materials and Methods

### Synthesis of PDA-indocyanine nanoparticles of various sizes

PDA was synthesized under alkaline conditions following previous reported method [10]. Dopamine hydrochloride (40 mg; Sigma-Aldrich, USA) was dissolved in 20 ml of tertiary distilled water (TDW), and a 1 N of NaOH solution was slowly added until the dopamine solution reached a pH of 9, 9.2, 9.7, and 10. After stirring at room temperature for approximately 24 h, the mixture was centrifuged at 21,206*g* for 20 min and washed with TDW. After performing 3 cycles of centrifugation, the produced PNs were dissolved in TDW and stored at 4 °C. PN solutions of 1 mg/ml were adjusted to pH 2 and mixed with a solution in which ICG was dissolved in TDW at a ratio of 2:1 (w/w). After stirring for 1 h, the ICG/PNs were purified by centrifugal washing 3 times at 21,206*g* to remove excess ICG. The obtained ICG/PNs were stored at 4 °C for further experiments.

### Characterization of PNs and ICG/PNs

PNs and ICG/PNs prepared at different pH values were characterized with respect to their size, zeta potential, ICG loading efficiency, absorbance, and fluorescence intensity. The size and zeta potential of the nanoparticles were measured by laser Doppler microelectrophoresis at an angle of 22° using an ELSZ-2000 instrument (Osaka, Japan). The ICG loading efficiency of the nanoparticles was calculated by measuring the absorbance of excess ICG after centrifugation using a Synergy HTX multimode reader and comparing it with the absorbance of free ICG. The morphology of ICG/PNs was observed using transmission electron microscopy (TEM). The fluorescence intensity of the ICG/PNs was measured using the Spectrum In Vivo Imaging System (IVIS) (PerkinElmer).

### Photostability and long-term storage stability test of ICG/PNs

The photostability of the nanoparticles was confirmed by measuring their absorbance after 808-nm NIR irradiation. For comparison, the absorbance of the PNs was also measured. Free ICG (0.8 mM) and ICG/PNs of various ICG/PN sizes were irradiated for 1, 3, and 5 min. The ICG/PN absorbance of each sample was measured using a Synergy HTX multimode reader. The degradation rate of ICG was calculated as the percentage of absorbance reduction at the maximal wavelength compared with that of nonilluminated ICG.

The long-term storage stability of ICG/PNs was confirmed by measuring ICG/PN their characteristics. Their size and zeta potential were measured using laser Doppler microelectrophoresis at different times.

### Cytotoxicity evaluation

The cytotoxicity of the nanoparticles was evaluated using a 3-(4,5-dimethylthiazol-2-yl)-2,5-diphenyltetrazolium bromide (MTT) assay. BALB/3T3 clone A31 cells (Korea Cell Line Bank, Seoul, Korea) were cultured in Dulbecco’s modified Eagle medium (DMEM), supplemented with 10% bovine calf serum and penicillin (100 IU/ml) and streptomycin (100 μg/ml). BALB/3T3 clone A31 cells were seeded in a 96-well plate at 0.5 × 10^4^ cells per well and cultured overnight. The next day, the cells were treated with nanoparticles at various concentrations in DMEM for 24, 48, and 72 h. Then, the cells were washed with phosphate-buffered saline, and 20 μl of MTT solution (10 mg/ml) was added to the prepared DMEM medium for another 4-h incubation. Next, the medium was removed, and MTT formazan crystals were dissolved in dimethyl sulfoxide and measured using a Synergy HTX multimode reader. Cell viability was calculated as the relative decrease compared with the absorbance of the control, which was considered 100% viable.

### Analysis of lymph node accumulation of nanoparticles

Lymph node accumulation of the ICG/PNs of various particle sizes was studied in BALB/c mice using fluorescence imaging. Fifty microliters of ICG/PNs (equivalent to 100 μg of ICG per mouse) from various nanoparticle samples were subcutaneously injected into the left hind foot pad of BALB/c mice. After 24 h, the IVIS was used to analyze the biodistribution of the popliteal lymph nodes closest to the footpad of the mouse.

A dose of ICG/PNs (equivalent to 100 μg of ICG per mouse) was subcutaneously injected into the left forefoot pads of BALB/c mice. After 24 h, the lymph nodes were fixed in 10% formalin, refrigerated, and frozen the following day. After freezing, they were cut into microsections (10 mm) using a microtome (Leica CM3050S, Leica, Nussloch, Germany) and stained with 4′,6-diamidino-2-phenylindole (DAPI). Fluorescence images were obtained using a light-emitting diode-based surface microscope (Leica Microsystems GmbH, Wetzlar, Germany) equipped with a Thunder system.

### Metastatic lymph node detection in orthotopic tumor model

For the establishment of a metastatic lymph node mouse model, subcutaneous injections of 1 × 10^5^ 4T1-Luc cells were administered to the left third mammary fat pads (situated in the upper left quadrant) of the BALB/c mice. Two weeks after tumor inoculation, the ICG/PNs, categorized into 4 distinct size groups, were subcutaneously administered to the left forefoot pads of BALB/c mice. Following a 24-h interval, 200 μl of luciferin solution (15 mg/ml) was injected, and bioluminescence imaging was conducted. Fluorescence imaging was performed using an IVIS.

Normal BALB/c mice and those with lymph node metastasis were subcutaneously injected with ICG/PNs into the left forefoot pad, receiving a dose of ICG/PN (equivalent to 100 μg of ICG per mouse). After a 24-h interval, the lymph nodes of the mice were fixed with 10% formalin and frozen in optimal cutting temperature medium for cryosectioning. The frozen lymph nodes were sectioned at 10 μm using a microtome. Following DAPI staining, the cells were observed using an light-emitting diode-based surface area microscope equipped with a Thunder system.

### Measurement of the retention rate of ICG/PNs in lymph nodes

The BALB/c mice were divided into 3 groups (*n* = 3), and 50 μl of ICG or 80-nm ICG/PN (equivalent to 100 μg of ICG per mouse) was administered to each mouse via subcutaneous injection into its left hind foot pad. The biodistribution of the popliteal lymph nodes proximal to the mouse footpad was analyzed using the IVIS at 24, 48, and 72 h after injection.

### Evaluation of systemic toxicity

The assessment of systemic toxicity involved monitoring various parameters, including mouse behavior, mortality rates, and hematological and blood biochemical parameters. Briefly, mice were intramuscularly injected with 50 μl of 80-nm ICG/PN (equivalent to 100 μg per mouse) or a 5% glucose solution as a control. Seven days after injection, a comprehensive evaluation was conducted, which included analyzing the general symptoms, alterations in body weight, and hematological and blood biochemical parameters. These evaluations were conducted in collaboration with Chemon (Korea) in response to specific requests.

### Statistical analysis

Student’s *t* test and one-way analysis of variance (ANOVA) using Student–Newman–Keuls tests were used for statistical evaluation of the experimental data. All statistical analyses were performed using GraphPad Prism 9.0 (a *P* value of less than 0.05 was considered statistically significant).

## Results

### Characterization of ICG/PN synthesized at different particle size ranges

**Fig. 1.**
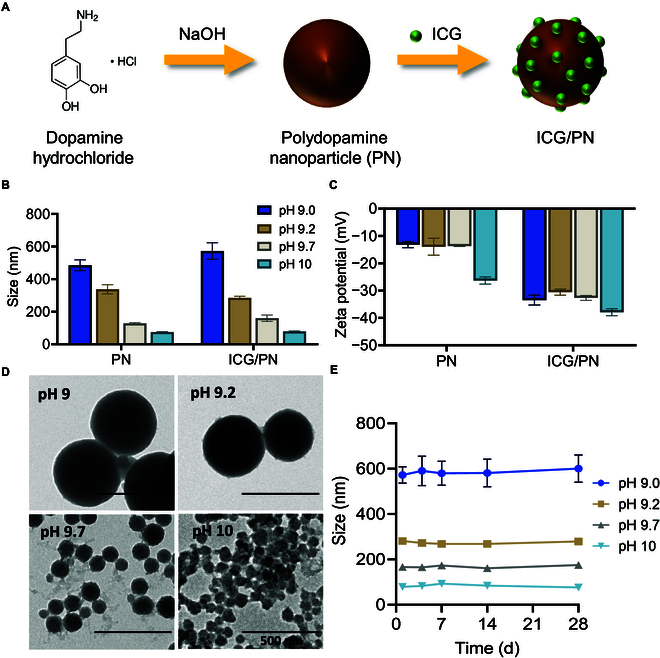
Characterization of nanoparticles. (A) PNs are synthesized by self-polymerization of dopamine hydrochloride and loaded with ICG. (B) Particle size and (C) zeta potential of PNs and ICG/PNs synthesized under various pH conditions during polymerization. (D) Morphologies of ICG/PNs synthesized under pH values of 9, 9.2, 9.7, and 10 as revealed by TEM. (E) Particle size of ICG/PNs measured over a period of 28 d.

PNs were synthesized via self-polymerization under alkaline conditions and atmospheric oxygen. NaOH was used as the reagent for pH control because of its ease of by-product removal and environmental friendliness compared to other reaction conditions requiring organic solvents such as ammonium and ethanol [11]. The particle size of the PNs can be adjusted by controlling the pH of polymerization. We chose pH values of 9, 9.2, 9.7, and 10 to synthesize PNs with sizes of 496.3 ± 34.8, 337.6 ± 28.0, 127.4 ± 12.7, and 73.7 ± 2.5 nm, respectively. ICG was loaded on purified PNs via π–π stacking interaction, yielding the corresponding ICG/PNs with sizes of 593.4 ± 99.6, 296.6 ± 24.7, 160.1 ± 19.8, and 78.6 ± 2.3 nm (Fig. 1A). The sizes are slightly increased compared to the respective original PN sizes (Fig. 1B). To screen for the size-dependent activities of ICG/PNs, we categorized them into 600-nm ICG/PN, 300-nm ICG/PN, 160-nm ICG/PN, and 80-nm ICG/PN, corresponding to their approximate average size. Because of its negative charge, ICG reduced the zeta potential of the final ICG/PN products for each particle size. The highly negative charge of the ICG/PNs, in the range of 30 to 40 mV, guarantees long-term colloidal stability (Fig. 1C). These zeta potential shifts confirm the absorption of ICG on the PN surface. TEM imaging revealed that the ICG/PNs were spherical and their size decreased with an increase in pH (Fig. 1D). By monitoring the colloidal stability of the ICG/PNs, the high colloidal stability of the particles was confirmed because the particle size showed negligible change during the 28-d storage course (Fig. 1E).

### ICG loading efficiency and performance of lymph node tracking

**Fig. 2.**
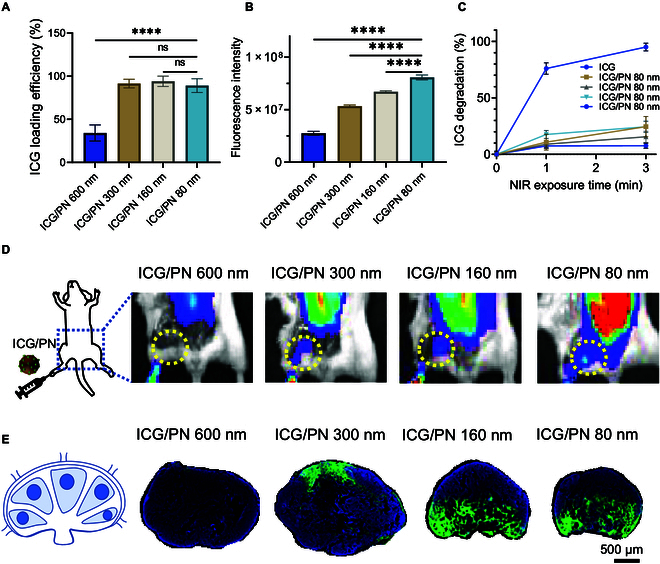
Fluorescence efficiency, photostability and lymph node tracking performance of ICG/PNs. (A) ICG loading efficiency of ICG/PNs. (B) Fluorescence intensity of ICG/PNs. (C) Degradation level of ICG loaded on ICG/PNs at various particle sizes. Samples were exposed to NIR laser for 3 min. (D) In vivo fluorescence images of mice treated with ICG/PNs with different particle sizes via foot pad injection. The sentinel lymph node locations were marked with a dashed circle. (E) Fluorescence images of sentinel lymph nodes isolated from treated mice, 24 h after injection. ****P < 0.001. ns, not significant.

The absorption of ICG by the PNs was measured based on loading efficiency. At an ICG:PN feeding ratio of 1:2 (w/w), 600-nm ICG/PN was able to carry 34.1 ± 9.3% of the fed ICG, and decreasing the size improved the drug absorption efficiency by up to 91.4 ± 5.0%, 94.0 ± 6.0%, and 89.0 ± 8.0% for ICG/PN 300 nm, 160-nm ICG/PN, and 80-nm ICG/PN, respectively (Fig. 2A). The fluorescence intensity of the ICG/PNs was highest for 80-nm ICG/PN, whereas 160-nm ICG/PN and 300-nm ICG/PN were less fluorescent at similar loading efficiencies (Fig. 2B). The photostability of ICG in the free or bound form on PNs of various sizes was evaluated by exposure to an NIR laser for 3 min. Free ICG was degraded by 75.9 ± 0.1% after 1 min and degraded almost completely, that is, by 95 ± 3.4%, after 3 min. In contrast, the degradation of ICG in 600-nm ICG/PN, 300-nm ICG/PN, 160-nm ICG/PN, and 80-nm ICG/PN was 7.7 ± 2.3%, 24.4 ± 4.9%, 15.5 ± 5.0%, and 24.5 ± 9.0%, respectively. These data indicate that the PNs were able to protect ICG from degradation at all tested size ranges (Fig. 2C). The biocompatibility of ICG/PN evaluated on a BALB/3T3 clone A31 fibroblast indicated material safety (Fig. S1).

In vivo fluorescence imaging confirmed that the ICG/PNs were deposited at sentinel lymph nodes 24 h after they were administered via foot pad injection. Fluorescence signals were detected in the sentinel lymph nodes of all mice treated with various ICG/PN particle sizes; however, the intensity varied among the groups (Fig. 2D). To examine the size-dependent deposition of ICG/PNs in the lymph node substructures, we performed fluorescent histological analysis. The 600-nm ICG/PN slightly stained the subcapsular sinus of the lymph node, whereas 300-nm ICG/PN stained both the subcapsular sinus and the small paracortex area. In contrast, 160-nm ICG/PN and 80-nm ICG/PN stained the paracortex and medulla through the subcapsular sinus (Fig. 2F).

### Metastatic lymph nodes mouse model

**Fig. 3.**
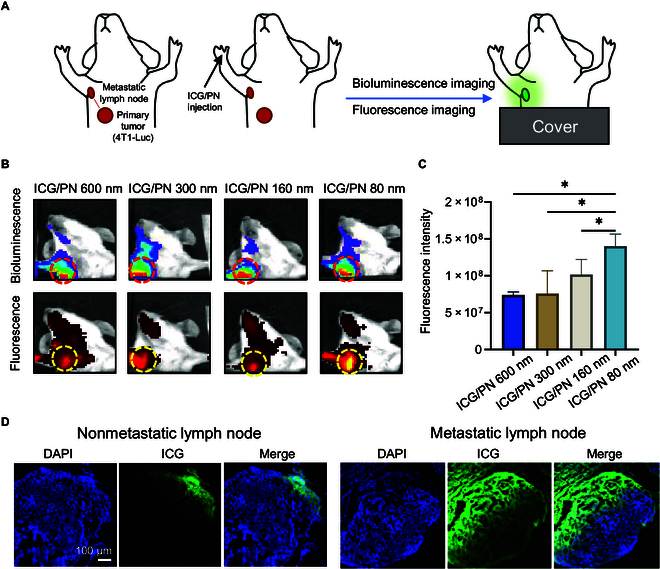
In vivo tracking of metastatic lymph nodes. (A) The metastatic lymph node model was established using the 4T1-Luc orthotopic tumor model. The location of metastatic lymph nodes was investigated using bioluminescence imaging. Lymph node distribution of ICG/PNs was detected using sequential fluorescence imaging. (B) Bioluminescence images and fluorescence images of mice treated with ICG/PNs with various particle sizes. The lymph node locations were indicated with dashed circles. (C) Fluorescence intensity measured at metastatic lymph nodes. (D) Histological imaging of nonmetastatic lymph node and metastatic lymph node of mice treated with 80-nm ICG/PN. *P < 0.05.

The hypothesis that ICG/PNs can track metastatic lymph nodes was supported by monitoring the fluorescence signal in mice bearing luciferase-active 4T1 tumors. First, the metastatic lymph nodes were examined using bioluminescence imaging. Fluorescence imaging was sequentially performed to track the ICG/PN signals (Fig. 3A). Alignment of the bioluminescence of luciferin derived from tumor cells and the fluorescence signal of the ICG/PNs indicated the deposition of ICG/PNs at the metastatic lymph nodes (Fig. 3B). The fluorescence intensity was observed to be higher in the metastatic lymph nodes of mice that were administered smaller-sized ICG/PNs, with 80-nm ICG/PN exhibiting the strongest fluorescence signal (Fig. 3C). The feasibility of lymph node metastasis tracking by 80-nm ICG/PN was evaluated by comparing the fluorescence signals of nonmetastatic and metastatic lymph nodes. A stronger signal was observed across the metastatic lymph nodes, whereas only a partial area of the nonmetastatic lymph nodes was fluorescent (Fig. 3D).

### Lymph node retention kinetics of 80-nm ICG/PN

**Fig. 4.**
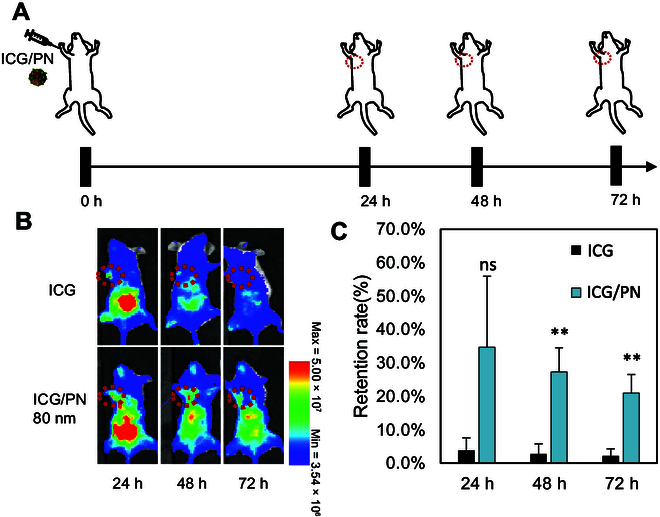
Lymph node retention of ICG/PN. (A) Schematic for the injection schedule to evaluate the retention kinetics of ICG/PNs in the lymph nodes. (B) Biodistribution of ICG and 80-nm ICG/PN over time. (C) Graph of fluorescence signal retention rate in lymph node of mice treated with ICG and 80-nm ICG/PN at various detection times. **P < 0.001.

The retention of 80-nm ICG/PN in the lymph nodes was evaluated by comparing their clearance kinetics with those of the lymph nodes with free ICG injection. Fluorescence images were captured at 24, 48, and 72 h (Fig. 4A). Notably, the fluorescence of 80-nm ICG/PN persisted in the lymph node area for the entire 72-h duration, whereas the signal in free ICG-treated mice was only detectable for 24 h. 80-nm ICG/PN exhibited a relatively sustained fluorescence, whereas ICG displayed a significant decline (Fig. 4B and C).

### In vivo toxicity test

**Fig. 5.**
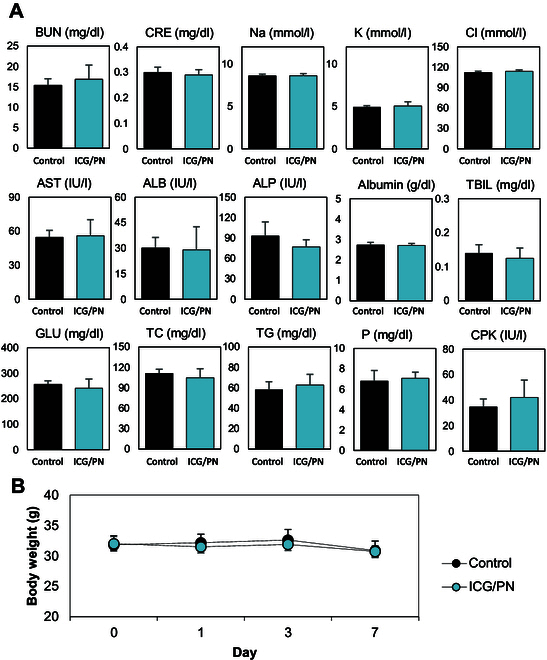
Clinical biochemical test. (A) Biochemical parameters of mice including ALP, AST, ALT, GLOB, ALB, TBIL, GLU, TCHO, CRE, BUN, Ca, Na, K, Cl, TG, IP, and CPK. (B) Body weight of treated mice observed over a 7-d period.

The assessment of toxicity following the administration of ICG/PNs involved a comprehensive analysis including biochemical tests, hematological tests, and body weight measurements. Seven days after treatment, alkaline phosphatase (ALP), aspartate aminotransferase (AST), alanine aminotransferase (ALT), globulin (GLOB), albumin (ALB), total bilirubin (TBIL), glucose (GLU), total cholesterol (TCHO), creatinine (CRE), blood urea nitrogen (BUN), calcium ion (Ca), sodium ion (Na), potassium ion (K), chloride ion (CI), triglyceride (TG), inorganic phosphorus (IP), and creatine phosphokinase (CPK) showed no statistical difference compared to control group, as depicted in Fig. 5A. In the hematological analysis, an array of parameters related to blood composition were analyzed. Red blood cells, hemoglobin, hematocrit, mean hematocrit, mean hemoglobin, mean corpuscular hemoglobin concentration, hemoglobin distribution width, platelets, mean platelet volume, white blood cells, lymphocytes, monocytes, eosinophils, basophils, and large unstained cells were all within the normal range. While neutrophils showed a slight increase, these changes remained within acceptable ranges according to the hematological standards for BALB/c mouse strain, based on values listed in database of Charles River Laboratories (Table S1). Furthermore, body weight served as an additional criterion to assess the overall physiological impact of the ICG/PN treatment. Remarkably, the injection of 80-nm ICG/PN did not result in any substantial change in the body weight of the treated mice when compared to the body weight of the 5% GLU-treated control group over the 7-d observation period. Collectively, these results suggested that ICG/PN administration did not adversely affect the overall health and growth of the treated mice (Fig. 5B).

## Discussion

In recent years, various efforts have been invested to study the structure of PDA and the process of its formation [12,13]. PDA is formed via the self-polymerization of dopamine under alkaline or oxidizing conditions. The mechanism of PDA formation has been discussed previously. To simplify the PDA production process, dopamine was oxidized to quinone under alkaline conditions, leading to the formation of 5,6-dihydroxyindole (DHI) through a nucleophilic reaction. Subsequently, DHI polymerized into oligomers, ultimately resulting in PN formation. This process involves the generation of semiquinone radicals from DHI and its oxidized form, indole-quinone, which further polymerizes via a radical-induced cross-linking pathway [14]. Several factors influence the particle size of PNs, including the monomer concentration, reaction temperature, and pH. Notably, an increase in the reaction pH is associated with a reduction in particle size. This correlation is attributed to the increased kinetic constant of polymerization, which leads to a faster nucleation rate and the formation of smaller particles [15]. In this study, we controlled the reaction pH and used pH values of 9.0, 9.2, 9.7, and 10. PNs with sizes of 600, 300, 160, and 80 nm (Fig. 1B and E) were synthesized. The PN candidates with size smaller than 80 nm were not included in this study because of several factors. First, we chose the simplest method for PN synthesis to facilitate large-scale synthesis. With this approach, synthesizing and utilizing smaller nanoparticles are not practical because of the requirement for extremely high centrifugation speeds to gather a sufficient particle yield. Furthermore, smaller nanoparticles may encounter issues of colloidal instability stemming from their high surface-to-volume ratio and faster Brownian motion, potentially leading to increased aggregation. Second, to synthesize ultrasmall PNs with sizes less than 50 nm, stabilizing PNs with polyethylene glycol was reported in a study [16]. This additional modification step complicates the entire process and may alter the PN’s surface properties notably. This aspect is crucial to consider in our study, as it could consequently affect ICG loading and cellular interactions, rendering them incomparable with other unmodified PN candidates in the study. Therefore, the diverse range of PN sizes from 80 to 600 nm enabled us to conduct a comprehensive assessment of size-dependent factors, including drug-loading ability and lymph node distribution of the PN carrier.

ICG is absorbed onto the PNs by interacting with the particle surface. This interaction is driven by hydrogen bonding between the abundant catechol/amine groups on the PN backbone and the ICG molecules. In addition, π–π stacking likely played a role in strengthening the interaction between the PNs and ICG. Exposure of the negative charge from the sulfonate groups of the ICG surface simultaneously reduced the negative surface charge of the PNs, thus ensuring prolonged colloidal stability of ICG/PNs (Fig. 1C and E). Of note, because of the presence of abundant hydroxyl phenol groups on PDA, the negative charge of PDA caused by pH increase could be due to the deprotonation of hydroxyl phenol in the presence of high [OH–] level. 80-nm PN was synthesized at pH 10, which has the highest [OH–] concentration. Therefore, the highest deprotonation degree at this pH yielded the most negative charge in 80-nm PN compared to the other groups (Fig. 1B).

Among the tested particle sizes, a smaller PN particle size yielded a higher ICG-loading efficiency, which may be attributed to an increase in the absorption area when the particle size was reduced. As depicted in Fig. 2A, ICG/PN with sizes of 300 nm, 160 nm, and 80 nm demonstrated similar and nearly maximal loading efficiency at a PN:ICG feeding ratio of 2:1 (w/w) compared to less than 50% loading efficiency of 600-nm ICG/PN. This finding suggests that ICG/PN with sizes of 300 nm, 160 nm, and 80 nm could accommodate a comparable amount of ICG. A previous study on the size-dependent impact of Au nanoparticles on fluorescence quenching suggested that larger particle dimensions lead to greater resonance energy and electron transfer [17]. This scenario may apply to PNs because an increase in particle size leads to a reduction in ICG fluorescence. Consequently, despite exhibiting similar loading efficiency, ICG/PN particles with a size of 80 nm exhibited the highest fluorescence emission compared to the other larger particle sizes. A marked drawback of ICG as a fluorescent contrast agent is its poor photostability. Loading ICG onto the PN surface mitigated photodegradation, which is likely attributable to the resonance energy transfer from the closely positioned ICG molecules to the PN backbone. The shielding effect of PNs against ICG photodegradation holds promise for extending the duration of ICG detection using fluorescence imaging.

Particle size plays a pivotal role in optimizing lymphatic drug delivery [18]. Larger nanoparticles (>100 nm in diameter) and microparticles tend to be trapped in the interstitial matrix at the injection site or require phagocytosis by tissue dendritic cells or Langerhans cells for cell-mediated delivery to the lymph nodes. In our study, 600-nm ICG/PN exhibited a barely detectable fluorescence signal at the lymph node site, suggesting virtual entrapment at the injection site. Conversely, 300-nm ICG/PN displayed a distinct fluorescence signal in the subcapsular sinus and partial areas of the paracortical sinuses. This observation suggests that a portion of the particles of this size drained through the initial lymphatic vessels and were eventually captured by subcapsular sinus macrophages. Previous research has indicated that small nanoparticles (10 to 250 nm in diameter) are absorbed by lymphatic vessels, diffuse into the lymph nodes, and target lymph-node-resident dendritic cells. In addition, materials can enter through the 0.1- to 1.0-μm gaps of the subcapsular sinus, allowing diffusion into the B cell follicle [19,20]. In line with these findings, 160-nm ICG/PN and 80-nm ICG/PN were delivered deep into the paracortex and medullary sinuses, resulting in substantial fluorescence staining across a major portion of the lymph node. Consequently, our findings indicate that smaller nanoparticles have an enhanced ability to penetrate lymph nodes.

The potential to differentiate between nonmetastatic and metastatic lymph nodes was also evident with small nanoparticles. Luciferase gene-carrying transformed 4T1-Luc cells were subcutaneously inoculated into the third mammary fat pad of mice to establish a lymph node metastasis model consistent with previous reports (Fig. S2) [21]. As cancer cells proliferate in the mammary fat pad tissue, they metastasize in the following sequence: the accessory axillary lymph nodes, proper axillary lymph nodes, mandibular lymph nodes, and accessory mandibular lymph nodes [22]. Indeed, metastasis of cancer to both the axillary and mandibular lymph nodes was confirmed 2 weeks after inoculation by the luciferase signal (Fig. 3B).

In cases of cancer metastasis and disease, the subcapsular sinus macrophage layer detaches from the inflamed lymph nodes, enabling pathogens to infiltrate the lymph node parenchyma or spread systemically [23]. Despite the general limitation of not having direct access to the lymph node parenchyma, 80-nm ICG/PN can still be transported to this region [24]. The prolonged retention of the fluorescence signal in the lymph nodes was likely a result of the slow clearance of ICG/PNs. This unique feature is beneficial for extending the window for lymph node detection during surgery. Moreover, differences in fluorescence intensity between regular and metastatic lymph nodes can offer valuable information to surgeons during lymph node resection, thereby enhancing the precision of surgical removal.

## Conclusion

In summary, our study on the synthesis of ICG-loaded PNs revealed the size-dependent effects that are crucial for drug delivery and imaging applications. By controlling the reaction pH, PNs with sizes ranging from 600 to 80 nm were synthesized. Smaller PNs exhibited superior ICG loading efficiency and fluorescence intensity, demonstrating their potential for use in fluorescence imaging. Our study highlights the critical role of particle size in lymphatic delivery, with smaller PNs demonstrating enhanced penetration into the lymph nodes and improved detection of lymphatic metastasis. This study contributes to the optimization of PNs for diverse therapeutic applications such as drug delivery, vaccine delivery, and immune therapy based on lymph node targeting.

### Ethics approval and consent to participate

All animals were maintained and used in accordance with the Guidelines for the Care and Use of Laboratory Animals of the Institute of Laboratory Animal Resources, Institutional Animal Care and Use Committee of Seoul National University (Seoul, Republic of Korea; approved animal experimental protocol number, SNU-210106-4)

## Data Availability

Data will be made available on request.
