# Supplementary material for: Size-Dependent Effect of Indocyanine Green Nanoimaging Agent for Metastatic Lymph Node Detection
Source: Biomater Res. 2024 Apr 15;28:0022. doi: 10.34133/bmr.0022 (PMC11018487; doi:10.34133/bmr.0022)
Supplement: Supplementary 1 — Fig. S1 Table S1 [file bmr.0022.f1.docx]

***Supplemental Materials***

***Size-dependent effect of indocyanine green nano-imaging agent for metastatic lymph node detection***

Quoc-Viet Le ^a,1^, Sungtaek Kang ^b,1^, Jaeseong Lee ^b^, Hyeseon Park ^b^, Jeong Gil Sun ^b^, Jaiwoo Lee ^c,^*, Gayong Shim ^b,^*

^a^ Faculty of Pharmacy, Ton Duc Thang University, Ho Chi Minh City, Vietnam,

^b^ School of Systems Biomedical Science and Integrative Institute of Basic Sciences, Soongsil University, Seoul, 06978, Republic of Korea,
^c^ College of Pharmacy and Research Institute of Pharmaceutical Sciences, Seoul National University, Seoul 08826, Republic of Korea

^1^ Quoc-Viet Le and Sungtaek Kang contributed equally to this study

* Corresponding author (Tel: 82-2-820-0451)

E-mail: ljw1112@snu.ac.kr or shim@ssu.ac.kr

***
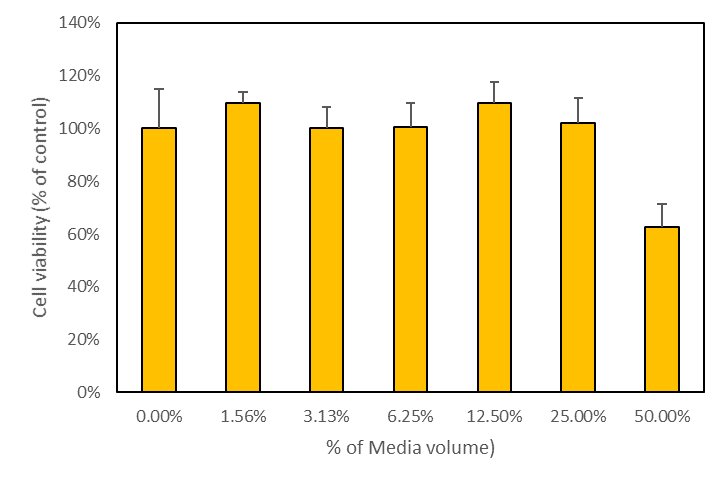
***

**Fig. S1. Cytotoxicity of ICG/PN 80 nm.**

Cell viability of BALB/3T3 clone A31 cell upon exposure to ICG/PN 80 nm at various concentrations.

| Parameters | **RBC** | **HGB** | **HCT** | **MCV** | **MCH** | **MCHC** |
| --- | --- | --- | --- | --- | --- | --- |
|  | (10^6^/µL) | (g/dL) | (%) | (fL) | (pg) | (g/dL) |
| 5%Glucose | 8.29 | 13.18 | 40.88 | 49.36 | 15.88 | 32.18 |
| ICG/PN | 8.43 | 13.38 | 42.4 | 50.32 | 15.9 | 31.62 |
| Parameters | **HDW** | **PLT** | **MPV** | **WBC** |  |  |
|  | (g/dL) | (10^3^/µL) | (fL) | (10^3^/µL) |  |  |
| 5%Glucose | 2.03 | 1182.6 | 6 | 2.82 |  |  |
| ICG/PN | 2.07 | 1199.8 | 6.2 | 3.74 |  |  |
| Parameters | **NEU** | **LYM** | **MONO** | **EOS** | **BASO** | **LUC** |
|  | (%) | (%) | (%) | (%) | (%) | (%) |
| 5%Glucose | 11.96 | 81.82 | 1.5 | 4.22 | 0.08 | 0.42 |
| ICG/PN | 15.2 | 79.22 | 1.42 | 3.82 | 0.06 | 0.32 |

**Table S1. Hematological test of control and treated mice**

Red blood cells (RBC), hemoglobin (HGB), hematocrit (HCT), mean hematocrit (MCV), mean hemoglobin (MCH), mean corpuscular hemoglobin concentration (MCHC), hemoglobin distribution width (HDW), platelets (PLT), and mean platelet volume (MPV), white blood cells (WBC), neutrophils (NEU) lymphocytes (LYM), monocytes (MONO), eosinophils (EOS), basophils (BASO), large unstained cells (LUC).
